# A Test Battery for Measuring Individual Cognitive Ability: A Brief Practical Tutorial

**DOI:** 10.5964/ejop.17163

**Published:** 2026-05-29

**Authors:** Maria Lialiou, Martine Grice, Petra B. Schumacher

**Affiliations:** 1 Department of German Language and Literature I, University of Cologne, Cologne, Germany; 2 I*f*L – Phonetics, University of Cologne, Cologne, Germany; University of South Wales, Cardiff, United Kingdom

**Keywords:** inhibition, processing speed, digit span, individual variability, attention, test battery

## Abstract

This tutorial provides a comprehensive, open-source collection of tasks, adapted and designed to measure key cognitive functions including inhibition, working memory, and processing speed. While a substantial body of research in experimental psychology focuses on these cognitive functions, many of the corresponding tasks are not readily accessible in one place — at least to the best of our knowledge. This lack of availability can create barriers for researchers seeking to investigate cognitive variability, necessitating additional time and effort to collate, design or code tasks that may already exist in some form. To address this issue, the present tutorial aims to make a battery of cognitive tasks freely available to the research community. By sharing these tools, we hope to enhance reproducibility in studies of cognitive variability and reduce the redundancy of repeatedly creating similar tasks from scratch. This resource is intended for researchers with an interest in exploring individual differences in cognitive and linguistic performance, offering a practical and efficient solution for accessing standardized tasks. Overall, this tutorial serves as a valuable contribution to the field, providing accessible tools to facilitate research, promote consistency across studies, and save researchers significant time and resources.

Individual differences in cognitive and language processing can stem from diverse cognitive functions. Although a large number of studies in experimental psychology and linguistics have investigated individual differences in language processing, and cognition more generally, through various tasks, these tasks are not accessible in one place in the form of a test battery. This limited and distributed availability poses challenges for researchers investigating cognitive variability, often requiring significant time and effort to design or code similar tasks independently. To our knowledge, the only recent open-source battery is the *Individual Differences in Language Skills* (IDLaS-NL), developed at the Max Planck Institute for Psycholinguistics in Nijmegen (Hintz et al., 2024; 2025). While highly valuable, this battery includes no measures of executive functions such as inhibition — which are central to our research — and uses different processing speed tasks. The battery does contain an auditory digit span task, but with limited control over the intonation patterns on the digits, another aspect that is central to our research. Furthermore, it is available in Dutch for native speakers and a German version is currently being validated (Bethke et al., 2025), but an English version is not yet released. Our battery, though smaller in scope, complements this resource by targeting these gaps.

This tutorial^1^1This article presents cognitive assessment methods originally developed as part of the first author's doctoral dissertation and later published in the book *Prosody and Attention Orienting* (Language Science Press). Although task descriptions in this article overlap substantially with those in the book, the book emphasises the question of intonation processing, whereas this article provides a focused tutorial on how to investigate sources of individual variability in behaviour, regardless of the question addressed. We have obtained permission to reuse this material and provide proper attribution throughout. addresses the accessibility gap by providing researchers with freely available cognitive tasks in one place. Specifically, the present test battery is a collection of open-source adapted versions of the flanker (Eriksen & Eriksen, 1974) and odd-man-out tasks (Frearson & Eysenck, 1986) implemented in *OpenSesame* (Mathôt et al., 2012), as well as an adapted auditory version of a digit span task (Wechsler, 1987) implemented in *SoSci Survey* (Leiner, 2024). This test battery measures individual cognitive variability in terms of inhibitory control (flanker task), processing speed (odd-man-out task), and working memory capacity (digit span task).

Although a wide range of tasks has been used across studies to assess inhibition, processing speed, and working memory capacity, a systematic comparison and comprehensive understanding of the relative strengths of these tasks is still lacking, and the available literature provides sparse findings. Accordingly, we do not claim that the tasks we selected represent the optimal measures, but rather well-established and practical choices within this context. For example, the flanker task is a standard paradigm for inhibitory control. It provides a sensitive measure of the ability to suppress interference from distracting (not conflicting) stimuli, which is central to the construct of inhibition. Further, compared to alternatives (e.g., Stroop, Stop-Signal), the flanker task offers a relatively simple design, and minimizes verbal or language-related confounds (Eriksen & Eriksen, 1974). The odd-man-out task was chosen because it has been frequently used to index visual processing speed in a standardized and time-efficient way, emphasizing rapid perceptual discrimination rather than strategy use (e.g., Diascro & Brody, 1994; Frearson & Eysenck, 1986). Finally, the digit span task was included as one of the most widely used and psychometrically validated measures of working memory capacity, offering strong normative data and cross-study comparability (e.g., Frischkorn et al., 2022).

By sharing these tools, the tutorial seeks to enhance reproducibility in research and reduce redundancy in task creation, allowing researchers to focus more on the analysis and interpretation of their findings. This collection is particularly relevant for those interested in exploring variability in cognitive and linguistic performance, offering practical, standardized resources to facilitate a wide range of investigations. The current cognitive battery has been made freely available online for use at Lialiou et al. (2025).^2^2This repository also shares an adapted version of the Simon task, which is another measure tapping into inhibitory abilities. Researchers are welcome to use this task as well. This task is not described in detail here, as the authors did not include it in their experimentation.

## The Tasks

### The Flanker Task

**Figure 1:**
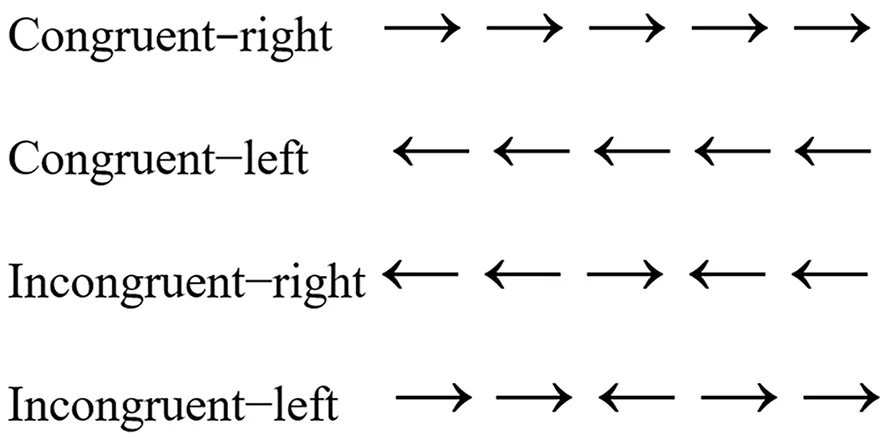
Examples of Items in the Flanker Task Across Different Conditions

To evaluate inhibitory control, the test battery incorporated an arrow-based adaptation of the flanker task (Anwyl-Irvine et al., 2020; Eriksen & Eriksen, 1974). The following task description is adapted from Lialiou (2025, pp. 106–107). In this task, a sequence of five arrows appears horizontally, in the middle of the screen. The core task is to indicate the direction of the central arrow in this sequence by pressing on the keyboard “S” for left or “K” for right. To minimize cognitive demands, participants are permitted to rest their fingers on the designated response keys. The task includes congruent trials, in which all arrows face in the same direction, and incongruent trials, in which the central arrow points in the opposite direction to the flanking arrows (see Figure 1). The task requires responses to be as fast as possible without compromising accuracy.

The task comprises 96 experimental trials, evenly divided between congruent and incongruent conditions (48 trials each). Within the congruent condition, 24 trials consist of uniformly rightward-pointing arrows, while the remaining 24 trials consist of uniformly leftward-pointing arrows (see Figure 1, top rows). Within the incongruent condition, 24 trials present rightward-pointing flankers with a leftward-pointing central arrow, while the remaining 24 trials present leftward-pointing flankers with a rightward-pointing central arrow (see Figure 1, bottom rows).

The task starts with a practice block containing 12 trials, followed by the experimental task, which is organized into four blocks with 24 trials each. Within each block, there is an equal proportion of the four trial types (congruent-right, congruent-left, incongruent-right, incongruent-left). Both trial order within blocks and sequence of blocks are fully randomized.

**Figure 2:**
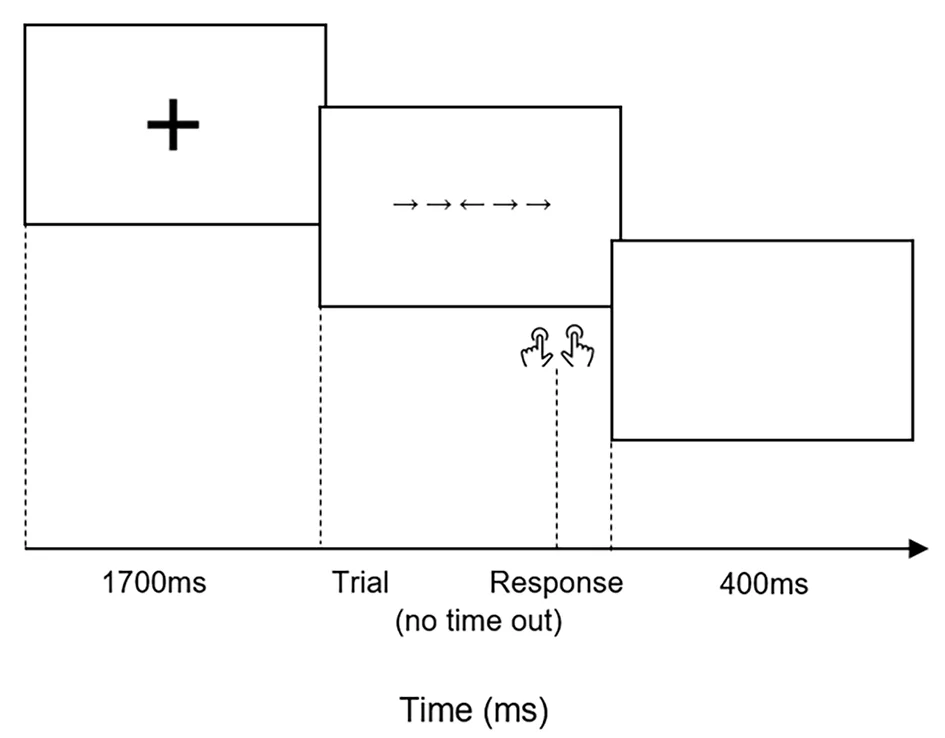
Overview of the Trial Structure in the Flanker Task

Each trial begins with the presentation of a fixation cross for 1700 ms, followed by the arrow array. The arrow array remains on the screen until participants press one of the two designated response keys (“S” for left or “K” for right). Trials finish with a 400 ms inter-trial interval during which a blank screen is shown. See Figure 2 for a schematization of the time course. The full task duration is approximately five minutes.

### The Odd-Man-Out Task

In the present test battery, processing speed is assessed using an adapted version of the odd-man-out task (Frearson & Eysenck, 1986). In the original version, participants saw a set of three stimuli and decided whether the first or the third stimulus deviated the most from the central one in terms of spatial proximity. In the current battery, following Diascro and Brody (1994), the original task was redesigned to increase complexity.

The present adapted task (see also Lialiou, 2025, pp. 107–109) manipulates two levels of task difficulty using three hexagonal figures organized into one of two odd-man-out formats: the spatial arrangement of the stimuli (hereafter, *spatial condition*) and the physical characteristics of the “odd-one-out” stimulus (hereafter, *gap condition*).

**Figure 3:**
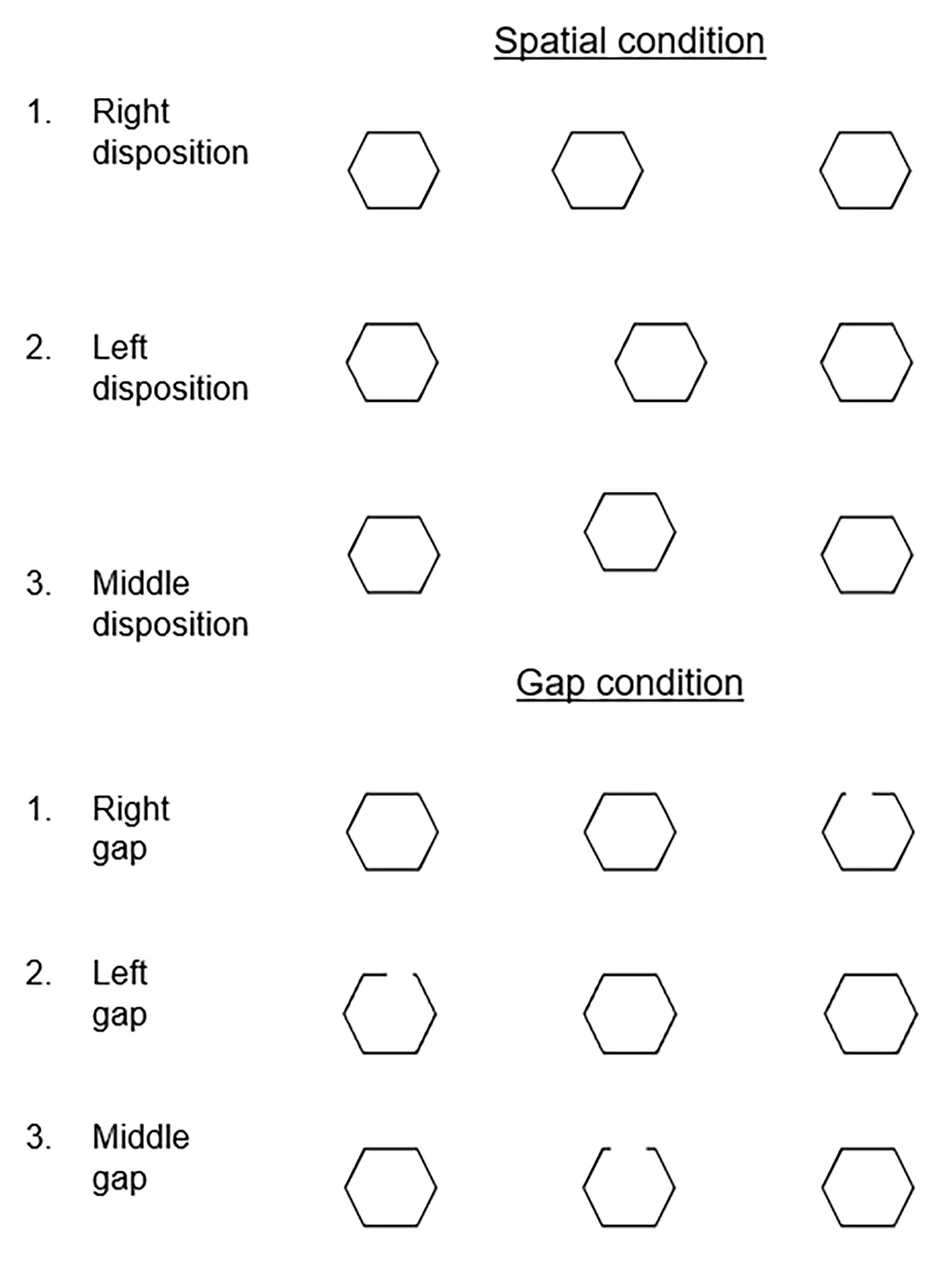
Illustration of Stimulus Configurations in the Odd-Man-Out Task *Note.* The upper panels correspond to spatial condition placements of the “odd-one-out”, and the lower panels correspond to missing-element positions of the “odd-one-out” in the gap condition.

In the condition targeting the spatial arrangement, the three hexagons are aligned horizontally, with one stimulus positioned farther away from the other two, thus constituting the “odd-one-out” item. As illustrated in the upper panels of Figure 3, this displaced stimulus can appear in one of three positions (left, center, or right). In the gap condition, all hexagons are evenly spaced horizontally. However, the “odd-one-out” stimulus is defined by a missing segment on its upper edge. As shown in the lower panels of Figure 3, the hexagon containing the gap could likewise occur in any of the three possible positions.

**Figure 4:**
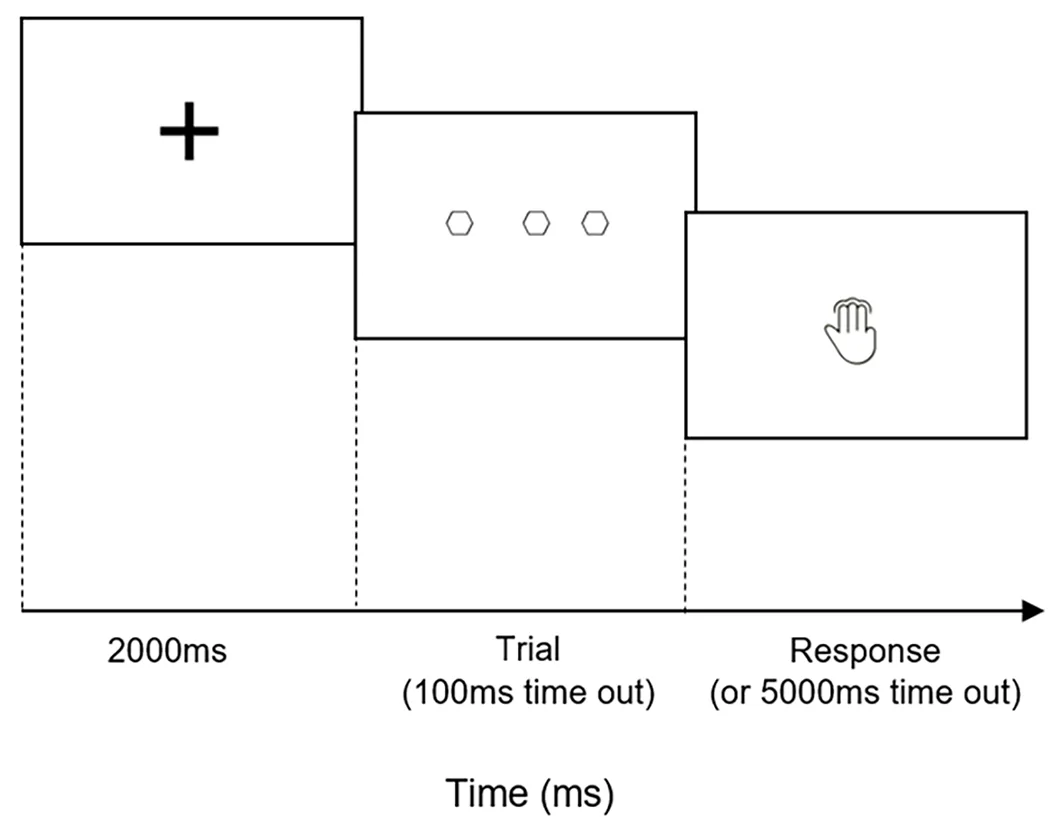
Overview of the Trial Structure in the Odd-Man-Out Task

The core task is to indicate the hexagon that is the “odd-one-out”, namely the one that differs from the others. The responses are recorded by pressing the following buttons on the keyboard: “1” if the “odd-one-out” stimulus is on the left, “2” if it is in the center, and “3” if it is on the right. The responses should be as fast and as accurate as possible. As described in the previous task, to minimize cognitive effort, participants may rest their fingers on the designated response keys (see Figure 4).

Participants are informed that they will begin with a practice block consisting of six trials. The main experiment comprises four blocks, each containing 30 trials — calculated as 5 items x 3 positions (right, left, middle) x 2 conditions (spatial, gap) — for a total of 120 experimental trials. Both item and block sequences are fully randomised. The trial structure is illustrated in Figure 4: each trial starts with a fixation cross displayed for 2000 ms, followed by the stimulus array, which appears for 100 ms. Immediately afterward, a blank screen is shown, which remains until the participant responds by pressing one of the three designated keys (“1”, “2”, or “3”) or until a 5000 ms time limit is reached. The entire task takes roughly five minutes to complete.

### The Auditory Digit Span Task

Finally, to incorporate measures of working memory in the current test battery, we adapted the digit span task derived from the WAIS-R Digit Span test (Wechsler, 1987). For this adapted version (see also Lialiou, 2025, pp. 109-110), stimuli recorded for a different experiment (Grice et al., 2024) were used. These stimuli are digits recorded with a flat fundamental frequency (F0) in German. Detailed instructions for recording similar stimuli in other languages are provided in the OSF repository.

**Figure 5:**
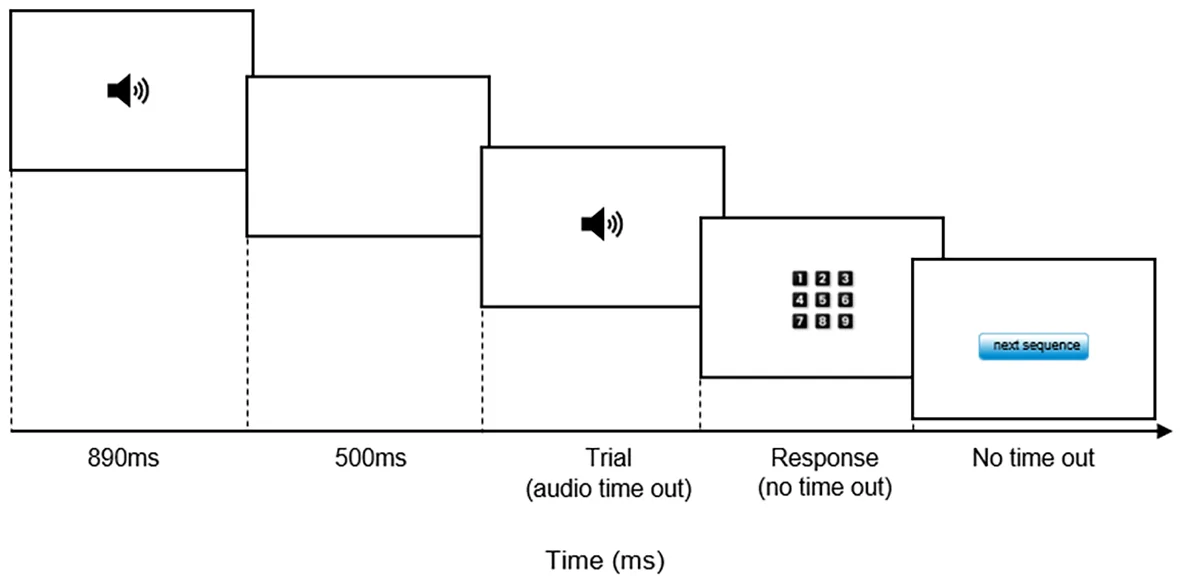
Overview of the Trial Structure in the Digit Span Task

This task presents auditory sequences of digits (1 to 9) that gradually increase in length. The task begins with a pair of three-digit sequences; after every pair of sequences of the same length, the next two sequences increase by one digit, continuing up to two nine-digit sequences, for a total of 14 experimental sequences. Participants are required to immediately recall each sequence in the exact order presented. Each sequence is played only once. Following the sequence, a numeric keypad appears on the screen, which is used by the participants to enter their responses by clicking the digits in the correct order. Every digit must be recalled; omissions are not allowed. A counter above the keypad indicates how many digits have been entered. Once the final digit is entered, participants proceed to the next sequence by clicking a “next” button. Each trial begins with an 890 ms beep, followed by 500 ms of silence before the digit sequence starts. The full task duration is approximately five minutes. The trial structure is depicted in Figure 5.

### Processing of the Output Data

For both the flanker task and odd-man-out task, each trial records participants’ performance in terms of accuracy — scored as correct (1) or incorrect (0) — and response time, measured in milliseconds. In our research, we focus on the role of individual variability in linguistic processing. Therefore, our analyses require incorporating individual variability as a covariate in the model, necessitating a single score per measure per participant. To achieve this, we propose the following method for processing the output data (see also Lialiou, 2025, pp. 111). However, researchers have access to the accuracy and reaction time data and may choose to process and analyze them differently.

To measure participants’ inhibitory control (Flanker task) and processing speed (Odd-Man-Out task), we employed the *efficiency measure* by Spilsbury et al. (1990). For the flanker task, an efficiency score can be calculated by dividing the number of correct responses on incongruent trials by the participant’s median response time (inhibition score = [correct incongruent responses] / [median RT]). A comparable efficiency measure can be computed for the odd-man-out task, where the number of correct trials is divided by the median response time to yield a processing speed score (processing speed score = [correct responses] / [median RT]).

Finally, in the Digit Span task, participants’ responses are recorded in the order they are recalled. Each participant’s digit span is determined by the length of the longest sequence correctly recalled before failing in two consecutive sequences. The smaller the span score, the smaller the WM capacity. In contrast, the larger the span score, the larger the WM capacity.

## Implementation of the Tasks

For the *OpenSesame* version tasks, there are two possible implementations: a local and an online one. Please, note that currently the Flanker and the Odd-Man-Out tasks are available with instructions written in German and English, while the auditory digit span task is available only in German. Therefore, if participants are speakers of a different language, the task instructions will need to be translated to ensure comprehension. For the auditory digit span task, detailed instructions for recording similar stimuli in other languages are provided in the OSF repository.

To set up the tasks locally, begin by downloading the free *OpenSesam*e software from https://osdoc.cogsci.nl/ and installing it on your computer. Next, obtain the *OpenSesame* code for the desired task from the OSF repository at Lialiou et al. (2025). The task folders are provided as .zip files, so you will need to extract them first. Once unzipped, open the experiment by clicking the *OpenSesame* icon, which will launch the experiment interface. From there, you can run the experiment directly.

For online implementation, start by creating a free account on https://mindprobe.eu/, a JATOS server supported by ESCoP and *OpenSesame*. Next, download the online version of the *OpenSesame* code for the relevant task from our repository; these are located within each task folder under “online implementation”. Do **not** unzip the downloaded files. Then, in the JATOS server, click “Import study,” select the downloaded .zip file, and upload it. The study will then appear in your list of studies and can be run immediately.

To run the *SoSci* Survey digit span task, create a free account at https://www.soscisurvey.de/ and import the HTML code from the repository as a new project. As noted above, the current version is in German only; guidance for adapting it to other languages is available in the OSF repository.

## Conclusion

This tutorial paper provided a collection of open-source tasks, designed and adapted to measure key cognitive functions such as inhibition, working memory, and processing speed. By providing freely available versions of the flanker, odd-man-out, and digit span tasks, along with detailed task descriptions and implementation guidance, we aim to facilitate access for researchers studying individual differences in cognitive and linguistic performance. The included suggestive analyses illustrate potential applications but are not intended as definitive methods. Beyond offering practical tools, this resource promotes reproducibility, efficiency, and transparency in cognitive and linguistic research. By making these tasks freely available, we hope to support the broader research community in exploring cognitive variability more effectively and consistently, reducing redundant effort, and fostering greater comparability across studies.

## Supplementary Materials

**Table:** 

Type of supplementary material	Availability/Access
Data	
No data provided.	—
Code	
No code provided.	—
Material	
Digit Span Task	Lialiou et al. (2025)
Flanker Task	Lialiou et al. (2025)
Odd-man-out Task	Lialiou et al. (2025)
Simon Task	Lialiou et al. (2025)
Study/Analysis preregistration	
Study was not preregistered	—
Other	
No other materials available.	—

## Data Availability

This current cognitive battery of tools is particularly relevant for those interested in exploring variability in cognitive and linguistic performance and has been made freely available online for use at Lialiou et al. (2025).
